# Development of a User-Friendly Pipeline for Mutational Analyses of HIV Using Ultra-Accurate Maximum-Depth Sequencing

**DOI:** 10.3390/v13071338

**Published:** 2021-07-11

**Authors:** Morgan E. Meissner, Emily J. Julik, Jonathan P. Badalamenti, William G. Arndt, Lauren J. Mills, Louis M. Mansky

**Affiliations:** 1 Molecular, Cellular, Developmental Biology & Genetics Graduate Program, University of Minnesota, Minneapolis, MN 55455, USA; schuc041@umn.edu; 2 Bioinformatics and Computational Biology Graduate Program, University of Minnesota, Minneapolis, MN 55455, USA; 3 Institute for Molecular Virology, University of Minnesota, Minneapolis, MN 55455, USA; ejjulik@gmail.com (E.J.J.); arndt255@umn.edu (W.G.A.); 4 Division of Basic Sciences, School of Dentistry, University of Minnesota, Minneapolis, MN 55455, USA; 5 University of Minnesota Genomics Center, University of Minnesota, Minneapolis, MN 55455, USA; jbadalam@umn.edu; 6 Masonic Cancer Center, University of Minnesota, Minneapolis, MN 55455, USA; 7 Department of Pediatrics, University of Minnesota, Minneapolis, MN 55455, USA

**Keywords:** HIV-1, HIV-2, sequencing, human immunodeficiency virus, mutation, sequence analysis

## Abstract

Human immunodeficiency virus type 2 (HIV-2) accumulates fewer mutations during replication than HIV type 1 (HIV-1). Advanced studies of HIV-2 mutagenesis, however, have historically been confounded by high background error rates in traditional next-generation sequencing techniques. In this study, we describe the adaptation of the previously described maximum-depth sequencing (MDS) technique to studies of both HIV-1 and HIV-2 for the ultra-accurate characterization of viral mutagenesis. We also present the development of a user-friendly Galaxy workflow for the bioinformatic analyses of sequencing data generated using the MDS technique, designed to improve replicability and accessibility to molecular virologists. This adapted MDS technique and analysis pipeline were validated by comparisons with previously published analyses of the frequency and spectra of mutations in HIV-1 and HIV-2 and is readily expandable to studies of viral mutation across the genomes of both viruses. Using this novel sequencing pipeline, we observed that the background error rate was reduced 100-fold over standard Illumina error rates, and 10-fold over traditional unique molecular identifier (UMI)-based sequencing. This technical advancement will allow for the exploration of novel and previously unrecognized sources of viral mutagenesis in both HIV-1 and HIV-2, which will expand our understanding of retroviral diversity and evolution.

## 1. Introduction

A high rate of viral mutation is a hallmark feature of human immunodeficiency virus type 1 (HIV-1) replication, leading to genetically diverse populations of the virus within a single host. This diversity promotes the development of antiviral drug resistance, cell tropism changes, and immune evasion, and contributes to the persistence of infection, not only within individuals, but across the globe [1]. The mutation rate of HIV-1 has been measured at 1.4–3.4 × 10^−5^ mutations per base pair (mut/bp) per replication cycle, with recent studies estimating that the mutation rate may be closer to 4.1 × 10^−3^ mut/bp per cell in vivo [2,3,4,5,6].

While the main driver of HIV-1 mutation is thought to be the low fidelity of the virally-encoded reverse transcriptase (RT) [7], a number of host factors can also contribute to viral mutagenesis. For example, the APOBEC3 (apolipoprotein B mRNA editing-enzyme catalytic polypeptide-like 3) family of DNA editing-enzymes have been identified as potent drivers of anti-HIV-1 mutagenesis through catalyzation of cytidine deamination reactions during reverse transcription [8]. This editing results in the generation of low levels of G-to-A hypermutants in the population which drives viral evolution, leading to disease progression, immune evasion, and failure of antiretroviral therapy [9,10,11,12,13]. Adenosine deaminase acting on RNA (ADAR) proteins have also been implicated in the mutagenesis of HIV-1 RNA during replication through the introduction of A-to-I editing in viral RNA, though the effects of this editing on infectivity remain unclear [14,15,16,17,18,19,20]. Other cellular factors, including cellular dNTP pools and cell type, also affect rates of retroviral mutation [21,22,23,24,25,26].

Compared with HIV-1, HIV type 2 (HIV-2) infection is characterized by lower viral loads within patients, lower rates of transmissibility between individuals, and slower progression to the AIDS disease state [27,28,29]. As a result, HIV-2 has remained largely geographically constrained to Western Africa, with decreasing prevalence in the population even as rates of HIV-1 infection in the region have increased [30]. Given the key role mutagenesis plays in driving HIV-1 progression, it has been hypothesized that lower rates of viral mutation may contribute to the attenuated disease phenotype of HIV-2 by limiting the diversification of the virus and reducing viral fitness [31]. Notably, HIV-2 was found to accumulate significantly fewer mutations during replication compared with HIV-1, exhibiting a significantly different mutation profile from HIV-1 with regards to both the rate and spectrum of observed mutations [20,31]. Overall, however, there has been a lack of appreciation in the differences in HIV-1 and HIV-2 mutagenesis and how their differing mutation rates affect viral diversity and fitness in human populations.

Advanced studies of HIV-2 mutagenesis have been limited due to high background error rates in traditional Illumina sequencing techniques, which are roughly similar to the intrinsic mutation frequency of HIV-2 [20,31]. Illumina sequencing error rates are generally estimated to be on the order of 10^−3^ mut/bp, but may be reduced to 10^−4^ or 10^−5^ mut/bp using both computational techniques and by adding unique molecular identifiers (UMIs) to sequence products [20,31,32,33]. However, the mutation rate of HIV-2 is similar to these intrinsic error rates, and even using sensitive UMI techniques, certain mutations (including transversions, insertions, and deletions) cannot be distinguished from background errors [20,31]. Some sequencing errors are generated during polymerase chain reaction (PCR) amplification, though most next generation sequencing (NGS) protocols utilize high-fidelity polymerases such as Phusion, *Pfu*, and Q5, which have error rates on the order of 10^−6^ and 10^−7^ mut/bp [34]. Additionally, C and A nucleotides are disproportionately mutated on NGS platforms and are more prone to substitution errors compared with G and T nucleotides [33,35]. Both C and A nucleotides are read in the red channel, suggesting that disturbances in the fluorescence, filters, or lasers may also result in erroneous base calls [35]. In particular, one study also reported that G was the nucleotide most frequently substituted in misread base calls, and A-to-G mutations were identified as the most commonly occurring errors in NGS [33,35]. It has been proposed that ADAR proteins may introduce low levels of A-to-G mutations in the HIV-1 and HIV-2 genomes [17,20]. Thus, advanced techniques are needed which reduce the intrinsic error rate of sequencing such that small changes in the HIV mutation rate, particularly for HIV-2, can be quantified at a higher signal-to-noise ratio and true mutations can be reliably distinguished from background errors.

A novel technique, maximum-depth sequencing (MDS), which reduces the theoretical error rate of Illumina sequencing to as low as 10^−8^ erroneous calls per bp was recently described [32]. This technique is different from conventional UMI-based sequencing techniques in that the UMIs are added directly to the starting template. This is done by initial digestion of the starting material with a restriction enzyme, resulting in a 3′ overhang adjacent to the region of interest (ROI) which serves as the substrate for polymerase extension directly from the genomic DNA (gDNA). The oligonucleotides used for extension contain a random 14-bp sequence, which serves as a UMI to generate consensus families that represent the composite of all reads resulting from a single starting template. Many mutations resulting from PCR and sequencing errors tend to occur randomly and will rarely be found in all members of a consensus read family. These random mutations can be excluded from the final consensus sequence for each read family, resulting in consensus sequences that more accurately reflect the true mutational profile of the starting gDNA template. Consensus family calling enabled by UMI-based sequencing, combined with the direct addition of the UMI to the start template, leads to significant reductions in the background error rate [32]. Although most studies of human somatic mutations, for example, do not require this level of sequencing sensitivity to warrant the added labor and costs associated with the MDS technique, the ultra-accuracy of MDS represents a much-needed advancement for mutational studies of HIV-1 and HIV-2.

Here we describe the adaptation of the MDS technique to quantify the rate and spectra of mutations across the HIV-1 and HIV-2 genomes, as well as the development of a user-friendly pipeline for the analysis of sequencing results. The rapid progression of sequencing technology has resulted in an increased need for bioinformatic expertise for data analysis. The development of a sequencing strategy that can be widely utilized therefore also requires the development of a user-friendly pipeline for data analysis, which is simple, accessible, and can be easily repeated. We created a pipeline that employs freely available, open-source software and tools for analysis of sequencing data and requires minimal inputs (i.e., a reference genome and the sequencing results) which can be used to analyze MDS data. This pipeline was subsequently developed into a Galaxy workflow to allow for improved access and replicability and is available for download online.

Using this adapted technique, the background error rate of HIV sequencing was reduced to approximately 1.6 × 10^−6^ mut/bp, which represents a 10- to 100-fold improvement over traditional Illumina sequencing methods [20,31,32,36,37]. The advanced sensitivity of this sequencing method will allow for studies of novel sources of HIV mutagenesis in both HIV-1 and HIV-2. The increased sensitivity provides a particularly significant advantage in the studies of mutagenesis of HIV-2, as well as cellular factors and small molecule mutagens that may cause more subtle changes in viral mutation patterns. The broad applicability of the adapted MDS technique, and the ease of data analysis facilitated by the generation of a user-friendly pipeline, will allow for research that will improve our understanding of novel sources of HIV mutagenesis.

## 2. Materials and Methods

### 2.1. Cell Lines and Plasmids

The adapted MDS technique was optimized using previously described HIV-1 and HIV-2 vectors (HIV-1 MIG and HIV-2 MIG) [38,39]. These vectors contain an *mCherry*-IRES-*EGFP* expression cassette cloned into the pNL4-3 and pROD10 viral backbones, respectively, and are deficient for *env* and *nef* expression, which limits viral replication to a single cycle. These plasmid vectors were used as negative controls to determine the background error rate of the adapter MDS technique. Because plasmids were not introduced to cellular or viral sources of mutagenesis, mutations in these samples represented only those resulting from errors in the sequencing pipeline, including those introduced during sample preparation, PCR amplification, and sequencing.

The HIV-1 and HIV-2 MIG constructs, pseudo-typed with the vesicular stomatitis virus glycoprotein (VSV-G), were used to generate viruses from 293T/17 cells using GenJet vII (SignaGen Laboratories). Cell culture supernatants were harvested, clarified by centrifugation at 1800× *g* rpm for 5 min, filtered through 0.2 µm filters, treated with DNaseI (10 U/µL) for 1 h and 30 min at 37 °C to remove residual plasmid, and aliquoted and stored at −80 °C. Viral titers in transducing units per milliliter (TU/mL) were determined by infecting U373-MAGI-CXCR4 cells in 24-well plates with varying volumes of virus and measuring the percentage of cells infected by flow cytometry, as has been described [40]. To generate samples for sequencing, U373-MAGI-CXCR4 cells seeded at a density of 1 × 10^6^ cells in 10 cm dishes and were infected at a multiplicity of infection (MOI) of 1 approximately 24 h later. Three days after inoculation, infected cells were harvested by trypsinization. An aliquot of cells was subjected to flow cytometry to validate the MOI, and the remainder were washed with phosphate buffered saline and frozen. Total gDNA was extracted from frozen cell pellets using the High Pure PCR Template Preparation Kit (Roche) and libraries were prepared, as described below.

### 2.2. Selection of Regions of Interest and Sample Preparation

A selection of ROIs spanning the HIV-1 and HIV-2 genomes were chosen based on their potential for mutagenesis, based in turn on previous studies of viral mutation profiles, as well as to provide optimal coverage of the HIV-1 and HIV-2 genomes. Primer sequences were designed using the HIV-1 and HIV-2 MIG vectors as templates, with special interest given to sequences which could be used to amplify additional constructs, including the pNL4-3.Luc.R^–^.E^–^ construct, obtained through the NIH AIDS Reagent Program, Division of AIDS, NIAID, NIH (contributed by Dr. Nathaniel Landau) [41,42], and the HIV-2 pGL-ANΔEnv-Luc and pGL-StΔEnvΔVpx-Luc constructs, kind gifts from Dr. Akio Adachi [43,44]. Primer sequences were also designed with consideration to appropriate restriction enzyme cut sites. Restriction enzymes were chosen based on the proximity of their cut sites to the ROI in question, as well as the uniqueness of the restriction enzyme recognition site within the gene, such that the enzyme would not cut within the region to be sequenced.

The ROIs, along with their corresponding primers and restriction enzymes, are described in Table S1. A total of 16 ROIs were chosen across both the HIV-1 and HIV-2 genomes. A portion of *int* was chosen for direct comparison with previous sequencing results, serving as a positive control for confirmation of expected trends in mutational patterns. Other regions were selected based on their potential as ROIs in studies of mutagenesis. For example, an ROI within the catalytic domain of *pol* was selected, as nucleotide changes in the dNTP binding site would be of particular interest in studies of small molecule mutagens. Amplicons were designed within regions of *vif* and *vpx*, which encode viral proteins which counteract host restriction factors that may influence viral mutagenesis. An ROI within the V3 loop region of *env* was also selected, as this region of the protein dictates co-receptor usage and cell tropism and has been found to be highly variable in quasi-species of HIV [45]. Additionally, ROIs within the highly structured sequences within the 5′ untranslated region (UTR) and rev response element (RRE) were chosen which may provide insights into editing by the ADAR family of proteins. A region within the *EGFP* reporter gene was selected for sequencing, which would allow for direct comparison of mutation profiles between HIV-1 and HIV-2.

**Figure 1:**
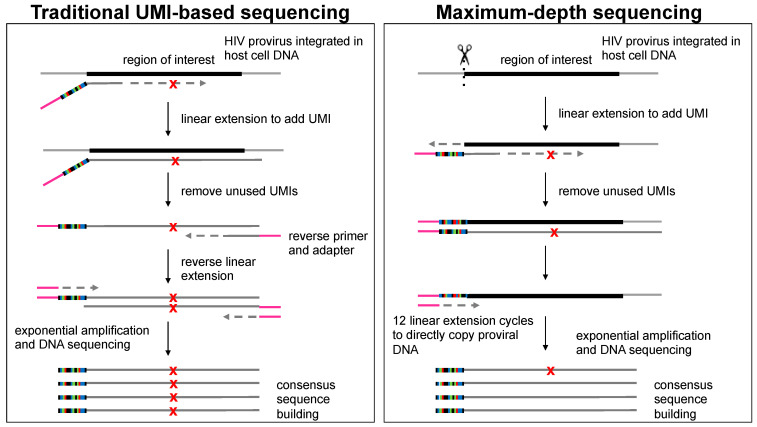
Schematic representations of traditional UMI-based sequencing and MDS. In traditional UMI-based sequencing, an initial linear round of PCR is used to copy the starting template and add a UMI. During this step, errors may occur at a low frequency dependent on the intrinsic error rate of the polymerase used, denoted by X. Exponential amplification from the tagged molecule results in the fixation of these errors within the read family, which then register as true virological mutations in the consensus sequence. In MDS, however, samples undergo an initial restriction enzyme digestion with a sequence-specific enzyme adjacent to the ROI, resulting in a 3′-OH overhang. A single round of linear amplification is performed which results in the addition of a UMI and the Illumina adapter to the sticky end of the gDNA molecule. The tagged gDNA molecule serves as the template for further rounds of amplification. Therefore, while polymerase errors may still appear in some reads, amplification occurs preferentially from the tagged starting gDNA molecule, such that reads with errors remain a minority of the read family and the consensus sequence remains a more accurate representation of the original starting template.

In addition to ROI-specific sequences, the primers contained Illumina adapters and UMIs (Tailed Primer Sequences in Table S1). A random 14-bp sequence included within the primer (denoted as “NNNNNNNNNNNNNN”) served as the UMI, which was unique to each amplified template. These UMIs were key to generating consensus sequences, as seen in Figure 1 and discussed below.

The technical and analytical aspects of the pipeline were first validated in a subset of six ROIs, encompassing the HIV-1 and HIV-2 5′ UTR, *int*, and RRE amplicons. The *int* amplicon was selected for direct comparison with previously published results. The 5′ UTR and RRE were included to ensure the efficiency of the pipeline across other regions of the HIV-1 and HIV-2 genomes.

The development of the MDS technique was described previously [32]. To prepare ROIs for sequencing, the gDNA template was digested using the appropriate restriction enzyme(s). An initial round of linear amplification was performed using the tailed primers. The 3′ overhang of the digested gDNA served as the substrate for extension during PCR amplification, resulting in addition of the UMIs directly to the starting template. In traditional sequencing techniques which utilize UMIs, an initial round of linear amplification is performed which copies the starting template and adds the UMI directly to the new DNA molecule. Errors can occur at a low frequency during this round of replication (dependent on the polymerase used in the reaction), which can then become fixed in the read family during subsequent rounds of exponential amplification. When read families are collapsed to create a consensus sequence, this mutation may be present in a majority of reads, thus erroneously being defined as a mutation which arose during viral replication. This results in higher error rates and an overestimation of the true mutation frequency. By adding the UMI directly to the starting molecule in the MDS pipeline, exponential amplification occurs directly from the starting gDNA and thus reduces the frequency at which polymerase errors become fixed within read families.

Unused UMI oligonucleotides were removed by exonuclease digestion. An additional 12 rounds of linear amplification were performed using an oligonucleotide with the same adapter sequence used in the initial Tailed Primer. Because an oligonucleotide with the complementary sequence was not included, the UMI-tagged gDNA molecule was exclusively amplified. Thus, mutations introduced as a result of polymerase errors during the initial rounds of linear amplification used to generate UMI-tagged molecules were not fixed in the population because they remain a minority of the tagged sequences in the pool of reads. Following linear amplification, an additional 15 rounds of exponential amplification were performed. A 1.5X AMPure XP bead cleanup (Beckman Coulter) was performed prior to and following a final PCR metabarcoding step, as described previously [46], and samples were submitted for library normalization and sequencing on the NovaSeq Illumina platform.

### 2.3. Estimation of Amplification Efficiency and Read Balancing

To address challenges observed in generating balanced representation of individual samples via standard methods, in which sequencing data were dominated by very small (one read) or very large (10,000+ reads) read families, a droplet digital PCR (ddPCR; Bio-Rad)-based balancing checkpoint was performed, which allowed for the estimation of starting sample copy numbers and amplification efficiency prior to sequencing. A 2 µL aliquot of the *EGFP* samples was removed before and after the initial linear amplification (i.e., UMI incorporation) step and was used to estimate the efficiency of linear amplification using ddPCR. Pre-tagged samples were amplified using ROI-specific primers (F: 5′-GAC GGC AAC TAC AAG ACC CGC-3′; R: 5′-GGC CAT GAT ATA GAC GTT GTG GC-3′). The same reverse primer (R) was used for the post-linear extension samples, along with an adapter-specific forward primer (5′-GTC GGC AGC GTC AGA TGT G-3′). Amplification efficiency, defined as the percentage of starting molecules that were tagged with a UMI during the initial linear amplification step, was calculated by dividing the number of tagged molecules by the number of starting molecules in the sample.

In subsequent rounds of sequencing, successful linear amplification and UMI tagging was confirmed by quantitative PCR (qPCR) using primers matching the sequence-specific portions of the forward and reverse tailed primers described in Table 1. This step served as a checkpoint to ensure efficient amplification of starting templates, which prevented significant biases in read family sizes and informed selection of an appropriate NovaSeq flow-cell to achieve desired sequencing yield without over-sequencing.

### 2.4. Generation of Consensus Sequences

**Figure 2:**
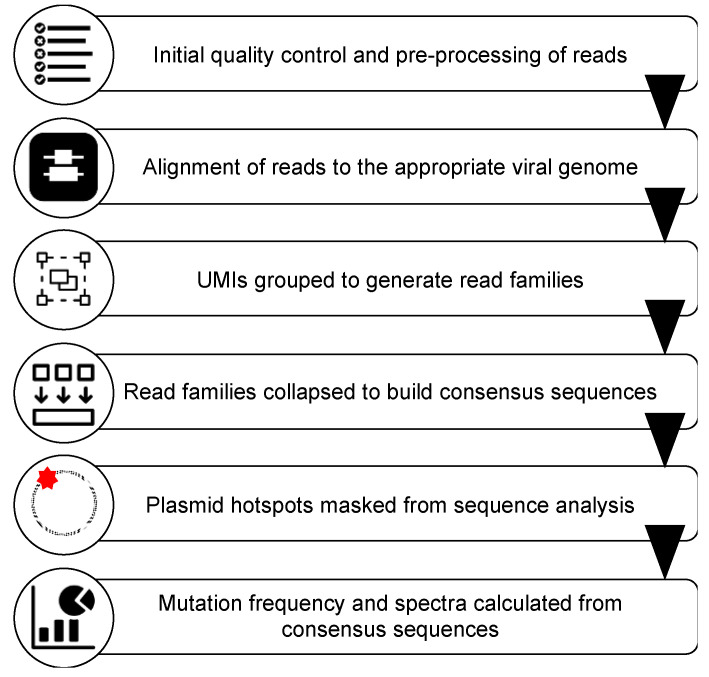
An overview of the pipeline developed for analysis of the MDS sequencing data. See text for details.

An overview of the pipeline developed for analysis of the data generated from MDS is shown in Figure 2. Sample quality was evaluated based on read count and levels of adapter contamination. High quality samples (i.e., those with at least 100,000 reads and less than 5% adapter contamination) were used for analyses. Illumina adapters and low-quality sequences were removed using Trimmomatic [47], with a quality score cutoff of 20. Fastq files were first converted to BAM files using FastqToBam from fgbio (Fulcrum Genomics) [48]. This conversion removed the first 14 bps (the length of our UMIs) of the sequence and preserved them in the resulting BAM file as the UMI associated with the read. Reads were then converted back to FASTQ format and were mapped to the appropriate viral genomes using BWA [49]. For mapping purposes, an approximately 200 bp region of the *3′ UTR* of each viral genome was masked to prevent misalignment of the *5′ UTR* amplicon to this region due to sequence similarity. Unique sample names were added as read group tags to all mapped and unmapped BAMs using AddOrReplaceGroups from Picard (Broad Institute) [50]. This allowed us to combine the UMI information in the unmapped BAM files with the sequence genomic location in the mapped BAM files using MergeBamAlignment (Picard), generating a final BAM file for each sample that could be used to generate consensus sequences.

Reads were then grouped by UMI to create read families using GroupReadsByUmi from fgbio. Grouping was performed using an adjacency strategy, which accounts for potential errors in sequencing that may occur within the UMI [51]. Using these read families, sequences were collapsed into a single consensus sequence using fgbio’s CallMolecularConsensusReads function. To be included in the consensus sequence, nucleotide calls had to appear in at least 75% of reads. Base calls that appeared in less than 75% of reads were considered errors, which may have arisen either during PCR amplification or Illumina sequencing, and were not included in the consensus sequence as a mutation which arose during viral replication. The minimum number of reads required to generate a consensus sequence was set to four reads. Consensus sequences were mapped back to the respective genome again using BWA. These consensus sequences represent the composite of all of the sequences observed in a given read family. Low frequency mutations have been removed, and the sequences reflect the most accurate representation of the profile of HIV-1 and HIV-2 pro-viral sequences in the starting sample.

### 2.5. Identifying Mutations

Mutations within consensus sequences were identified using mpileup (BCFtools). This tool generated a text file for each sample which contained the frequency and spectra of mutations at each position within a given sample. The depth of each base call within the consensus sequences represented the number starting templates in which a given nucleotide appeared. Alternative base calls, differing from the reference sequence, represented mutations. The types and depths of these nucleotide changes were used to determine the frequency and spectra of mutations at a given position and within an ROI. Analyses of mutations were performed using custom R scripts which identified plasmid hotspots and calculated the frequency and types of mutations observed within each sample (see below).

### 2.6. Masking of Plasmid Mutation Hotspots

Plasmid mutation hotspots, defined as positions that were preferentially mutated at a frequency found to be an upper outlier in the plasmid (negative control) data, have been previously reported in the literature [20,33,52]. To identify plasmid mutation hotspots, the mean percentage of alternative base calls was calculated at each position within plasmid samples for each ROI. Upper outliers were defined as those with greater than 1.5-times the interquartile range of the mean percent alternative base calls across all positions for that ROI. Positions in which two or more plasmid samples had upper outlier mutation frequencies were considered hotspots. However, because the background error rate was so low, for most ROIs, the interquartile range of the percent alternative base calls was 0, such that any position that had a single error was identified as a plasmid hotspot. To avoid unnecessary loss of data, only those positions in which the average number of mutations across all replicates was greater than one were considered true plasmid hotspots and masked from further analyses in both plasmid and gDNA samples in an ROI-dependent manner.

### 2.7. Analyses of Mutation Frequency and Spectrum

Following the masking of plasmid hotspots, the mutation frequency was calculated by dividing the total number of alternative base calls by the total number of bases sequenced for each sample. Differences in the mutation frequency were evaluated using multiple *t* tests. Mutation spectra were generated based on the distribution of alternative base calls within each ROI. Samples with a read depth less than 10,000 base pairs at each position were excluded from analyses. A minimum read depth of 10,000 base pairs was chosen as it represented roughly 10% of the average read depth achieved across all amplicons using the NovaSeq platform and was a sufficient depth to observe our expected mutation frequency, which was expected to be in the order of 10^−5^ mut/bp within a given amplicon.

## 3. Results

### 3.1. Amplification Efficiency

**Figure 3:**
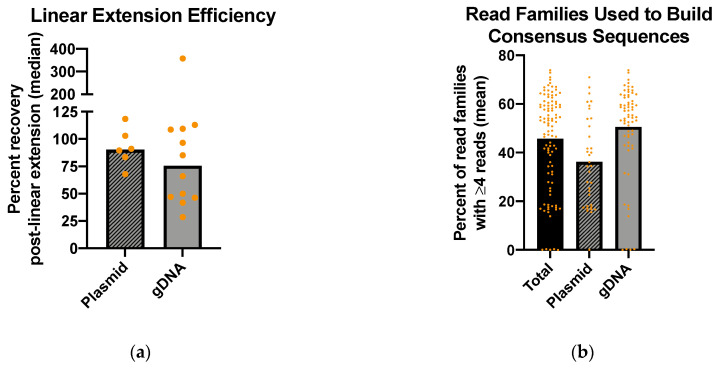
Amplification efficiency of the MDS pipeline. (**a**) Linear extension efficiency. The efficiency of linear extension was determined by dividing the number of tagged *EGFP* molecules following linear amplification by the total number of starting *EGFP* molecules, as measured using ddPCR. The median linear extension efficiency was 90.2% in plasmid samples and 75.5% in gDNA samples (*p* = 0.002). Replicates are marked as individual points; (**b**) Read families used to build consensus sequences. The efficiency of exponential amplification was evaluated by determining the percent of read families with 4 or more reads, which represented read families which would be used to generate consensus sequences. In total, an average of 45.7% of read families contained 4 or more reads (plasmid samples, 36.3%; gDNA samples, 50.6%).

The technical efficiency of the MDS pipeline was evaluated to ensure that robust capture of pro-viral sequences was observed. The efficiency of the linear extension step, in which UMIs were added to the starting template, was assessed using ddPCR quantification of EGFP before and after the initial linear amplification step (Figure 3a). The percentage recovery post-linear extension was estimated by dividing the estimated number of tagged molecules following the linear amplification step by the estimated number of starting molecules. In the plasmid samples, the median linear extension efficiency was 90.2%. The linear extension efficiency was slightly lower in the gDNA samples (median, 75.5%; *p* = 0.002), although there was considerable variability across samples. Collectively, though, these results suggest that the majority of starting molecules within a sample are efficiently tagged with a UMI for subsequent amplification and assessment of mutation frequency and spectra.

The efficiency of exponential amplification was assessed by evaluating the number of read families with four or more reads, which represented those reads which would be used to generate consensus sequences (Figure 3b). Across all samples, the mean percentage of read families with four or more reads was 45.7%. The percentage was lower in the plasmid samples (mean, 36.3%) than the gDNA samples (mean, 50.6%), although this difference was not significant. There were five samples in total which failed exponential extension (less than 1% of read families contained four or more reads), which occurred more frequently in the gDNA samples than in the plasmid samples (6.0% vs. 2.9%).

### 3.2. Plasmid Mutation Hotspots

**Figure 4:**
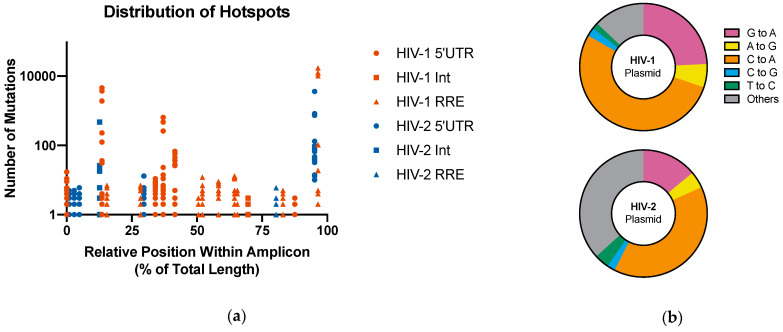
Analyses of background mutations in the MDS pipeline. (**a**) Distribution of plasmid mutation hotspots. The distribution of plasmid hotspots across ROIs was visualized by plotting the relative position of hotspots within the amplicon. A total of 23 sites across the 6 ROIs examined were determined to be hotspots. These occurred all across the amplicon and were not localized to the ends of the ROIs as may be expected due to artifactual errors. Mutation hotspots were dominated by transversion mutations, namely T-to-G mutations in HIV-1 samples (70.2%) and T-to-A mutations in HIV-2 samples (75.7%); (**b**) Relative frequency of background mutation types. Shown are pie charts depicting the frequency of mutation types of interest within the HIV-1 and HIV-2 plasmid samples, which were used as negative control to estimate the frequency and spectra of background errors. The frequency of background errors was 5.84 × 10^−6^ mut/bp in HIV-1 and 9.78 × 10^−6^ mut/bp in HIV-2 samples. The most frequently observed mutations in both plasmids were C-to-A mutations, which accounted for 53.1% and 39.3% of mutations in HIV-1 and HIV-2 plasmid samples, respectively. Mutations commonly associated with Illumina sequencing (e.g., A-to-G, C-to-G, and T-to-C mutations) were observed infrequently.

The number of plasmid mutation hotspots identified in the six ROIs examined are shown in Table 1. A single hotspot was identified in the HIV-1 and HIV-2 int ROIs as well as the HIV-2 RRE, which accounted for less than 0.5% of their respective ROIs. More hotspots were observed in the HIV-1 and HIV-2 5′UTR ROIs (*n* = 6 and 5, respectively) and the HIV-1 RRE (*n* = 9). These accounted for less than 5% of their respective ROIs. Consistent with previous results, hotspot mutations were predominantly transversion mutations [20]. In HIV-1, T-to-G transversion accounted for 70.2% of observed hotspot mutations. In HIV-2, 75.7% of hotspot mutations were T-to-A transversions. The relative position of the hotspot mutations within the amplicon was plotted for each ROI to assess whether positional biases may be occurring (Figure 4a). Hotspots occurred across the entire span of the amplicon and were not exclusively located at the ends of sequences, which are regions which typically contain sequencing errors. In samples with a single hotspot, none of the hotspots were identified within the first or last 10% of the amplicon.

### 3.3. Background Errors

HIV-1 and HIV-2 plasmids were used as negative controls to assess the frequency and spectrum of background errors in the adapted MDS pipeline. The mutation frequency was calculated by dividing the total number of base calls that differed from the reference (alternate base calls) by the total number of base pairs sequenced. Across all plasmid samples, the frequency of background mutations was 5.84 × 10^−6^ mut/bp in HIV-1 and 9.78 × 10^−6^ mut/bp in HIV-2. In both plasmids, there was a bias towards C-to-A mutations (Figure 4b). In HIV-1 plasmid samples, C-to-A mutations accounted for 53.1% of all mutations observed. These mutations represented a smaller percentage of observed mutations in HIV-2 plasmid samples (39.3%) but were still the most frequently occurring mutations. C-to-A mutations are characteristic of DNA damage and may be the result of oxidative stress which occurs during sample preparation [53,54]. Other mutations typically associated with Illumina sequencing, such as A-to-G, C-to-G, and T-to-C mutations, were observed at considerably lower levels, accounting for less than 6% of observed mutations in either the HIV-1 or HIV-2 plasmid sample.

### 3.4. Mutation Frequency and Spectrum in HIV-1 and HIV-2

**Figure 5:**
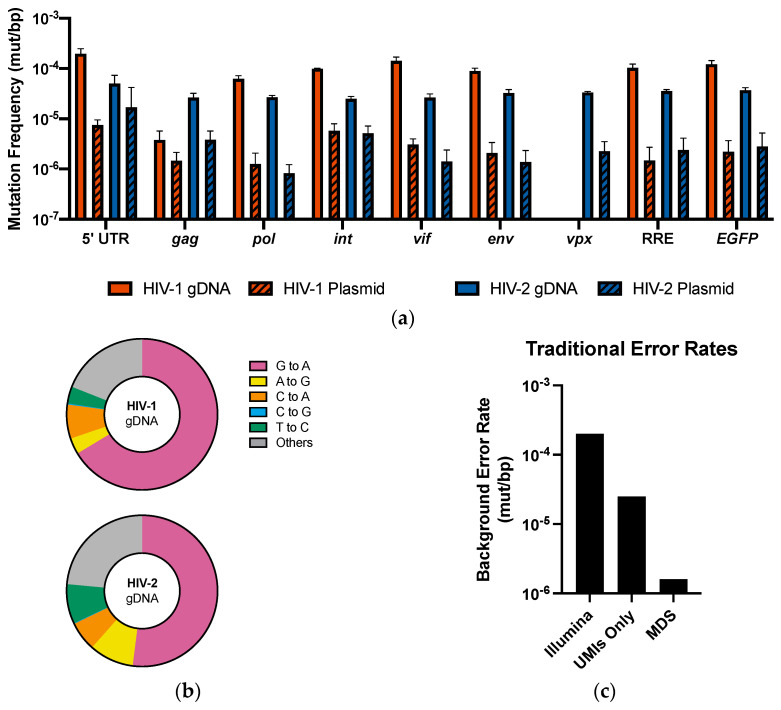
Mutation profile of ROIs in HIV-1 and HIV-2. The (**a**) mutation frequency and (**b**) mutation spectra were calculated for each of the 8 ROIs in HIV-1 and 9 ROIs in HIV-2. Across all ROIs, the mutation frequency was 9.35 × 10^−5^ mut/bp in HIV-1 and 3.14 × 10^−5^ mut/bp in HIV-2. The mutation frequency was significantly higher in gDNA samples than in plasmid samples for HIV-1 and HIV-2 across all amplicons (*p* < 0.05 for all). (**c**) Comparison of the background error rates in HIV-1 and HIV-2 plasmids using MDS with error rates previously observed with Illumina sequencing [31] and traditional UMI-based techniques [20].

Previous UMI-based sequencing efforts were used to explore the frequency and spectrum of mutations in the int gene [20]. To evaluate the improved efficacy of the adapted MDS technique, we further examined the mutation profile observed of int within the MDS dataset for comparison with previously published results. In the MDS dataset, mutations occurred at a frequency of 1.0 × 10^−4^ mut/bp in HIV-1 gDNA and 2.50 × 10^−5^ mut/bp in HIV-2 gDNA (Figure 5a). It is notable that the observed mutation frequency in HIV-2 gDNA is only slightly higher than the previously noted background errors rate in HIV plasmid samples using UMI-based sequencing of int (approximately 2.0 × 10^−5^) [20]. Using the adapted MDS technique, however, the background error rate was reduced almost five-fold, to 5.8 × 10^−6^ in HIV-1 plasmid samples and 5.1 × 10^−6^ in HIV-2 plasmid samples. For both viruses, the frequency of background errors was significantly lower than the frequency of mutations observed in gDNA (*p* < 0.000001 for both), suggesting that real mutations can be readily distinguished from background errors in sequencing.

For all 16 ROIs across both viruses, the background error rate of the adapted MDS technique was significantly lower than the mutation frequency observed in the gDNA samples (Figure 5a). Background error rates were similar across all ROIs, with the lowest rates observed in HIV-2 *pol* (8.3 × 10^−7^ mut/bp) and the highest background error rates observed in the HIV-2 5′UTR (1.7 × 10^−5^ mut/bp).

We observed a similar distribution of mutations across HIV-1 and HIV-2 ROIs with the adapted MDS technique as has been previously reported, in which the spectrum of mutations in HIV-1 is dominated by G-to-A mutations, which are observed less frequently in HIV-2 (Figure 5b) [20,31]. In HIV-1, G-to-A mutations accounted for approximately 66% of mutations observed, compared with approximately 52% of mutations in HIV-2. A-to-G mutations, which may be associated with ADAR editing, were observed at low levels in HIV-1 (3.5%), although appeared more frequently in HIV-2 samples (9.6%), which is consistent with previously published results [20]. Mutations associated with DNA damage and sequencing errors, including C-to-A and T-to-C mutations, were observed much less frequently than in the plasmid controls (seen in Figure 4b). As seen in the plasmid samples, errors frequently associated with sequencing errors represented a minority of the observed mutations.

Collectively, the background error rate observed across both HIV-1 and HIV-2 ROIs was about 1.6 × 10^−6^ mut/bp (Figure 5c). This represents an over 100-fold improvement over traditional Illumina sequencing rates, which have been previously estimated at 2 × 10^−4^ mut/bp [31,32,33]. Compared with traditional UMI-based sequencing, which has an error rate of approximately 2.5 × 10^−5^, this represents a 10-fold reduction in the background mutation frequency. These results suggest that the ultra-accuracy of the MDS pipeline represents a significant advantage over traditional sequencing methods for the detection of mutations within HIV-1 and HIV-2.

## 4. Discussion

Analyses of HIV mutagenesis, particularly in HIV-2, are challenged by intrinsically high error rates in Illumina NGS technologies, which occur at a frequency of about 10^−3^ to 10^−4^ mut/bp [31,32,33]. Although these error rates may be reduced using UMI-based sequencing, background mutations may still occur at a frequency that makes analyses of rare mutations difficult [20,31,32]. The addition of UMI tags directly onto gDNA targets in the MDS technique improves the sensitivity of UMI-based sequencing by reducing the rate at which errors may become fixed within read families [32]. Using this technique, error rates in Illumina sequencing may theoretically be reduced up to 1000-fold over conventional UMI-based sequencing techniques, depending on sequencing depth [32].

We adapted the MDS technique to create a pipeline for studies of HIV mutagenesis within 16 ROIs across the HIV-1 and HIV-2 genomes. These ROIs represented regions that are of interest to biologically diverse experimental questions, including studies of host restriction, small molecule mutagenesis, and viral evolution. Six ROIs in HIV-1 and HIV-2 (5′ UTR, *int*, and RRE) were selected for initial validation of the MDS pipeline. The *int* ROI was chosen for direct comparison with previously published UMI-based sequencing results from our lab [20], and the 5′ UTR and RRE were chosen to confirm the results of the pipeline across other regions of the genome.

We found that the technical aspects of the MDS pipeline exhibited relatively high efficiency. The initial linear extension step, which tagged the proviruses with UMIs, resulted in amplification of over 75% of starting templates in gDNA samples and 90% of starting templates in plasmid samples (Figure 3). Exponential extension was less efficient, with an average of 45.7% of read families containing four or more reads. These read families represent those from which consensus sequences are generated. Together, this indicates that an average of about 33% of plasmid starting templates and 38% of gDNA starting templates account for those analyzed for mutation frequency and spectra following sample preparation, sequencing, and read processing. For samples in which large numbers of cells can be readily infected at a high rate of infectivity, this would not be a limitation; however, this may present an obstacle in situations in which the acquisition of a high volume of starting templates is a challenge. These values may also represent an overestimation of the efficiency of amplification, as copy number calculations based on DNA concentration and gel electrophoresis of pre-linear extension products suggest that some amount of plasmid and gDNA samples remain undigested by the respective restriction enzyme.

Plasmid mutation hotspots have been previously identified in sequencing results and represent positions in negative control samples (plasmid) at which mutations accumulate at a disproportionately high frequency [20,33,52]. Hotspots may be caused by a variety of sources including PCR bias or issues specific to Illumina sequencing technology. In previous studies, up to 24 hotspots per ROI have been reported [31]. Of the initial six ROIs that were examined in our dataset, three ROIs contained only one plasmid hotspot, and the most observed in a single ROI was nine (Table 1). It is worth noting, however, that a less stringent approach for the identification of plasmid hotspots was used in the present study, in which a secondary filter was added that required mutations to occur at a frequency of greater than one per sample. This additional filter was added because the observed background error rate was so low that plasmid hotspots identified using previously described methods represented positions where any single mutation occurred within a plasmid sequence. This added filter therefore identified positions which more accurately met the definition of sites of mutational biases. This more liberal approach to identification of plasmid hotspots reduced the number of sites masked from downstream analyses.

Previous studies reported that the majority of plasmid hotspots were dominated by C-to-A or G-to-T transversions [20,31]. In contrast, the majority of the hotspot mutations observed using the MDS technique were T-to-G and T-to-A transversions for HIV-1 and HIV-2 plasmids, respectively. These mutations accounted for over 70% of the mutations in plasmid hotspots within their respective viruses. Plasmid hotspots were distributed across the full length of amplicons (Figure 4a). Errors related to sequencing are common at the ends of reads; however, we did not observe such a positional bias of plasmid hotspots, indicating that errors related to Illumina sequencing have been largely resolved. Hotspots may instead be attributable to PCR artifacts, such as biases in amplification efficiency or polymerase specificity.

In vector virus plasmid samples, which were used as negative controls to estimate the background mutation frequency, the most common mutations observed in both HIV-1 and HIV-2 samples were C-to-A mutations, which accounted for approximately 53% of mutations observed in HIV-1 plasmid samples and 39% of mutations observed in HIV-2 plasmid samples (Figure 4b). Previous studies have found that C-to-A mutations, as well as G-to-T mutations, are characteristic hallmarks of DNA damage due to oxidate stress during the sequencing process [53,54]. G-to-T mutations were observed at a much lower frequency than C-to-A mutations, occurring in less than 5% of HIV-1 and HIV-2 plasmid samples. However, the overabundance of C-to-A mutations in our dataset suggests that DNA damage may be a primary source of background mutations. C-to-A (as well as G-to-T) mutations are also the most frequently observed Q5 polymerase errors [34]. Although the addition of UMIs to the starting template is thought to effectively eliminate the occurrence of polymerase errors, these results suggest that some errors may still occur during the amplification of starting templates. Other mutations frequently associated with Illumina sequencing errors include A-to-G, C-to-G, and T-to-C mutations [33,35]. All of these mutations were observed at very low frequencies using the adapted MDS technique, accounting for no more than 6% of the background mutations observed.

Using the adapted MDS technique, we observed that background mutations occurred at a frequency of approximately 1.6 × 10^−6^ mut/bp, which represents an approximately 100-fold improvement in background error rates compared with traditional Illumina sequencing, and a 10-fold improvement compared with traditional UMI-based sequencing techniques (Figure 5c). Importantly, the background error rate was significantly lower than the mutation frequency observed across all amplicons (Figure 5a). This ensures that mutations detected within the genomes of HIV-1 and HIV-2, in particular, will be distinguishable from those erroneously introduced during sequencing. Even lower mutation rates may be achieved by increasing the depth of sequencing with MDS, which increases the number of reads within read families and improves detection of sequencing errors [32].

Additionally, although C-to-A mutations represented the majority of background errors observed in plasmid negative controls (Figure 4b), these mutations were observed infrequently in gDNA samples (Figure 5b), accounting for 7.3% and 6.3% of mutations observed in HIV-1 and HIV-2, respectively. Other mutations frequently associated with Illumina errors, including A-to-G and T-to-C mutations, were also infrequent, accounting for less than 10% of mutations observed across all ROIs.

In addition to the technical development and validation of the MDS technique, a sequence analysis pipeline has been established in this study for the complete processing of sequencing results from initial quality control to analysis of mutation frequency and spectra (Figure 2). This pipeline was developed using open-source, freely available software and can be accessed via a BASH script that can be edited to run in a Linux environment or as a Galaxy workflow. All of the tools used in the Galaxy workflow are in the Galaxy Tool Shed and can be installed to any Galaxy server. Pipeline scripts, custom R scripts and the Galaxy workflow are available for download here: https://github.com/ljmills/MDS_pipeline_HIV (accessed on 5 May 2021). This analysis pipeline represents a significant advancement for data analysis, and is much more user friendly to molecular virologists, as a strong background in computer science or bioinformatics is not required. The use of free tools and software increases the accessibility of this analysis pipeline, such that it can be performed on any personal computer. Furthermore, the use of a standardized protocol allows for the ready analysis and comparison of datasets. The discovery and monitoring of mutations that arise at low frequencies in HIV-1 and HIV-2 requires sensitive sequencing approaches, such as the adapted MDS pipeline. This sequencing strategy will have important implications for gaining a deeper understanding of the sources of HIV mutagenesis.

## 5. Conclusions

In conclusion, we have adapted the highly sensitive MDS technique to establish a pipeline for analyses of HIV-1 and HIV-2 mutagenesis which significantly reduces background error rates associated with the Illumina sequencing platform. This pipeline has been developed to use freely available software and tools and was designed to be accessible to molecular virologists. This pipeline was validated by comparisons with previously published analyses of the frequency and spectra of mutations in HIV-1 and HIV-2 and is readily expandable to studies of viral mutation across the genomes of both viruses. This experimental sequencing strategy will allow for the detection of rare mutations in the viral genome, particularly in HIV-2, which has a mutation frequency similar to the background error rate of previously described sequencing techniques. This technical advancement will allow for the exploration of novel and previously unrecognized sources of viral mutagenesis in HIV-1 and HIV-2, which fills a current knowledge gap in our understanding of retroviral diversity and evolution.

## Figures and Tables

**Table 1:** Number of plasmid mutation hotspots identified in each ROI.

ROI	Virus	Number of Hotspots	Percent of Amplicon
5′ UTR	HIV-1	6	3.02%
	HIV-2	5	2.44%
Int	HIV-1	1	0.44%
	HIV-2	1	0.44%
RRE	HIV-1	9	4.66%
	HIV-2	1	0.46%

## Data Availability

The data presented in this study are openly available in the BioProject database, accession number PRJNA736052.

## Supplementary Materials

The following are available online at https://www.mdpi.com/article/10.3390/v13071338/s1, Table S1: Primer design and amplicon information.
