# Supplementary material for: Development of a User-Friendly Pipeline for Mutational Analyses of HIV Using Ultra-Accurate Maximum-Depth Sequencing
Source: Viruses. 2021 Jul 11;13(7):1338. doi: 10.3390/v13071338 (PMC8310143; doi:10.3390/v13071338)
Supplement: Supplementary file 1 [file viruses-13-01338-s001.zip › viruses-1273355-supplementary.pdf]

ROI Number	Virus	Gene	F. Primer Sequence	F. Tailed Primer Sequence	R. Primer Sequence	R. Tailed Primer Sequence	Amplicon Size (bp)	Total Size (bp)	Restriction Enzyme
1	HIV-1	5'UTR	TCTGGTTAGACCAGATCTGAG	CAGCGTCAGATGTGTATAAGAGACAGNNNNNNNNNNNNNNNNTCTGGTTAGACCAGATCTGAG	CTTTCAAGTCCCTGTTCGG	GTCTCGTGGGCTCGGAGATGTGTATAAGAGACAGCTTTCAAGTCCCTGTTCGG	199	342	BmrI
2	HIV-2	5'UTR	CCCCACGCTTGCTTGCTTAA	CAGCGTCAGATGTGTATAAGAGACAGNNNNNNNNNNNNNNNNCCCCACGCTTGCTTGCTTAA	CTTCAAGTCCCTGTTCGG	GTCTCGTGGGCTCGGAGATGTGTATAAGAGACAGCTTCAAGTCCCTGTTCGG	205	348	HaeIII
3	HIV-1	Gag	CCTATCCCAGTAGGAGAAATCT	CAGCGTCAGATGTGTATAAGAGACAGNNNNNNNNNNNNNNNNCCTATCCCAGTAGGAGAAATCT	TACCTCTTGTGAAGCTTGC	GTCTCGTGGGCTCGGAGATGTGTATAAGAGACAGTACCTCTTGTGAAGCTTGC	176	319	BmrI
4	HIV-2	Gag	AGAAGATGGATCCAGATAGG	CAGCGTCAGATGTGTATAAGAGACAGNNNNNNNNNNNNNNNNAGAAGATGGATCCAGATAGG	GGATCTGTTTGTTCTGCC	GTCTCGTGGGCTCGGAGATGTGTATAAGAGACAGGGATCTGTTTGTTCTGCC	146	289	BccI
5	HIV-1	Pol (RT)	TGAGACACCAGGGATTAGAT	CAGCGTCAGATGTGTATAAGAGACAGNNNNNNNNNNNNNNNNTGAGACACCAGGGATTAGAT	GCTGCCCTATTTCTAAGTCA	GTCTCGTGGGCTCGGAGATGTGTATAAGAGACAGGCTGCCCTATTTCTAAGTCA	182	325	BcoDI
6	HIV-2	Pol (RT)	AACAATGCAGAACCAGGA	CAGCGTCAGATGTGTATAAGAGACAGNNNNNNNNNNNNNNNNAACAATGCAGAACCAGGA	CTGTCCTGTCACTAGCTAT	GTCTCGTGGGCTCGGAGATGTGTATAAGAGACAGCTGTCCTGTCACTAGCTAT	178	321	Hpy166II
7	HIV-1	Int	CCCAAGAAGAACATGAGAAATATC	CAGCGTCAGATGTGTATAAGAGACAGNNNNNNNNNNNNNNNNCCCAAGAAGAACATGAGAAATATC	TATCCACTGGCTACATGAAC	GTCTCGTGGGCTCGGAGATGTGTATAAGAGACAGTATCCACTGGCTACATGAAC	226	369	HaeIII
8	HIV-2	Int	CGCTCAGGAAGAACATGAAA	CAGCGTCAGATGTGTATAAGAGACAGNNNNNNNNNNNNNNNNCGCTCAGGAAGAACATGAAA	AATCCACTTGCAACATGTAC	GTCTCGTGGGCTCGGAGATGTGTATAAGAGACAGAATCCACTTGCAACATGTAC	228	371	Cac8I
9	HIV-1	Vif	ATCAGGGATTATGGAAAACAGAT	CAGCGTCAGATGTGTATAAGAGACAGNNNNNNNNNNNNNNNNATCAGGGATTATGGAAAACAGAT	CTAATTTAGCATCCCCTAGTGG	GTCTCGTGGGCTCGGAGATGTGTATAAGAGACAGCTAATTTAGCATCCCCTAGTGG	203	346	Sau3AI
10	HIV-2	Vif	TGGATAGTRGTTCCCACCT	CAGCGTCAGATGTGTATAAGAGACAGNNNNNNNNNNNNNNNNTGGATAGTRGTTCCCACCT	GTTCCAATATGCCTGTATCTC	GTCTCGTGGGCTCGGAGATGTGTATAAGAGACAGGTTCCAATATGCCTGTATCTC	204	347	BbsI
11	HIV-2	Vpx	GARCTYATTTTCCAGGTGTG	CAGCGTCAGATGTGTATAAGAGACAGNNNNNNNNNNNNNNNNGARCTYATTTTCCAGGTGTG	TAGACYAGACCTGGAGGG	GTCTCGTGGGCTCGGAGATGTGTATAAGAGACAGTAGACYAGACCTGGAGGG	212	355	AvaI
12	HIV-1	Env (V3)	CTGAACACATCTGTAGAAATTAATTG	CAGCGTCAGATGTGTATAAGAGACAGNNNNNNNNNNNNNNNNCTGAACACATCTGTAGAAATTAATTG	GGCATTCCATTTTGCTCTACTA	GTCTCGTGGGCTCGGAGATGTGTATAAGAGACAGGGCATTCCATTTTGCTCTACTA	159	302	AluI
13	HIV-2	Env (V3)	GTATATTGCACCCAATATGTAACTG	CAGCGTCAGATGTGTATAAGAGACAGNNNNNNNNNNNNNNNNGTATATTGCACCCAATATGTAACTG	TCCATTTGCCTTTGAACCAG	GTCTCGTGGGCTCGGAGATGTGTATAAGAGACAGTCCATTTGCCTTTGAACCAG	187	330	DdeI
14	HIV-1	RRE	ACAGGCCAGACAATTATTGT	CAGCGTCAGATGTGTATAAGAGACAGNNNNNNNNNNNNNNNNACAGGCCAGACAATTATTGT	AATGAGTTTTCCAGAGCAAC	GTCTCGTGGGCTCGGAGATGTGTATAAGAGACAGAATGAGTTTTCCAGAGCAAC	193	336	RsaI
15	HIV-2	RRE	GGGTTCTTGGGTTTTCTC	CAGCGTCAGATGTGTATAAGAGACAGNNNNNNNNNNNNNNNNGGGTTCTTGGGTTTTCTC	TGGTCCTGTAGGTACTTC	GTCTCGTGGGCTCGGAGATGTGTATAAGAGACAGTGGTCCTGTAGGTACTTC	218	361	BfaI
16	HIV-1 and HIV-2	EGFP	CGACGGCAACTACAAGACC	CAGCGTCAGATGTGTATAAGAGACAGNNNNNNNNNNNNNNNNCGACGGCAACTACAAGACC	CATGATATAGACGTTGTGGCT	GTCTCGTGGGCTCGGAGATGTGTATAAGAGACAGCATGATATAGACGTTGTGGCT	153	296	Hpy99I

N, any base; R, A or G; Y, C or T
